# “Walking with myself by my side” - non-medical use of Ketamine

**DOI:** 10.1192/j.eurpsy.2022.2148

**Published:** 2022-09-01

**Authors:** R. Vaz, J. Martins, A. Costa, J. Brás, R. Sousa, E. Almeida, J. Abreu, D. Teixeira, A. Marques, N. Gil, P. Carriço

**Affiliations:** 1 Centro Hospitalar Tondela-Viseu , Departamento De Psiquiatria E Saúde Mental, Viseu, Portugal; 2 DICAD, Centro De Respostas Integradas De Coimbra, Coimbra, Portugal

**Keywords:** Ketamine, Ketamine addiction, Ketamine use

## Abstract

**Introduction:**

Ketamine, synthesized in 1962 as phencyclidine derivate, is denominated a “dissociative anesthetic” because of its side-effects, such as dissociative episodes and psychotic-like symptoms, which have limited its applicability on clinical practice. Otherwise, in the last decades the non-medical use of ketamine has been growing and today is one of the most popular illicit substances consumed between adolescents and young adults.

**Objectives:**

Increasing the knowledge and understanding of the factors related to crescent use of ketamine and the experiences and consequences associated to its consumption.

**Methods:**

Clinical interview with patients diagnosed with ketamine use disorder and bibliographic research in Pubmed database using the terms “Ketamine use” and “Ketamine addiction”.

**Results:**

Pat et al. (2002) describes a clinical case of a young male, diagnosed with substance use disorders, specifically alcohol and cocaine use disorders, that started a treatment with ketamine. After the treatment, pleasant depersonalization experiences contributed to the development of patient’s ketamine dependence. Other patient’s reports confirm the association of ketamine use with psychedelic effects and dissociative episodes and pointed these effects as main reason for its consumption.

**Conclusions:**

The adverse effects that limited the medical use of ketamine are the same that promote its utilization with recreational purposes by adolescents and young adults in parties and nightclubs. About the ketamine dependence, the literature is scarce and doesn´t clearly identify a physical withdrawal syndrome, pointing only to a serious psychological dependence. Thus, with the crescent non-medical use of ketamine, it’s urgent to develop an intervention plan directed to this problem.

**Disclosure:**

No significant relationships.

